# Efficacy and safety of selenium disulfide in seborrheic dermatitis: what RCTs tell us?

**DOI:** 10.1097/MS9.0000000000003975

**Published:** 2025-09-30

**Authors:** Sarah Zulfiqar Khan, Nabiha Kashif, Syeda Fatima Ur Rehman, Hafsa Ajmal, Hafiz Sohail Ashraf, Muddassir Khalid, Fred Segawa

**Affiliations:** a Department of Medicine, Dow University of Health Sciences, Karachi, Pakistan; b Department of Medicine, King Edward Medical University, Lahore, Pakistan; c Carle foundation Hospital, Urbana, Illinios, USA; d Department of Medicine, Nishtar Medical University, Multan, Pakistan; e Department of Medicine, Makerere University College of Health Sciences, Kampala, Central Region, Uganda

Seborrheic dermatitis (SD) is a recurring inflammatory skin disorder affecting areas rich in sebaceous glands, with a global prevalence of 1–5%[1], primarily in adult men. Its pathogenesis is enmeshed with genetic predisposition, immune dysregulation, and *Malassezia* overgrowth. Although not life-threatening, its visible manifestations can significantly undermine self-esteem, perpetuating psychological distress and impairing overall quality of life. Randomized clinical trials (RCTs) are the gold standard for evaluating treatment outcomes. This editorial will encompass a thorough analysis of RCTs published comparing the efficacy, relapse prevention, and side effects of selenium disulfide (SeS_2_)-based regimens against treatments like ketoconazole, corticosteroids, and salicylic acid (SA). We did a systematic search from 2018 to 2025 and found several studies evaluating treatments for SD. In Massiot *et al*, SeS_2_ showed a decrease in erythema after 4 weeks of its use (*P* = 0.018) and following the maintenance phase (*P* = 0.006)[2] to its anti-fungal and antibacterial properties against *Malassezia* furfur and *Staphylococcus* species, respectively^[1,2]^.

Furthermore, a SeS_2_-based shampoo significantly reduced pruritus compared with its vehicle after initial topical corticosteroid (TCS) and SA therapy. After 8 weeks, 76.2% of patients in the SeS_2_ group reported no pruritus (vs 29.2% initially), with greater relief than the vehicle group (88 vs 67%). This suggests that beyond its active ingredient, shampoo’s formulation aids pruritus relief (*P* < 0.05) by interacting with scalp physiology, contributing to sustained symptom reduction[2]. SeS_2_ also shows comparable efficacy to other treatments like zinc pyrithione and ketoconazole^[3–5]^. According to Fernandes-Melo *et al*, by day 28, SeS_2_ and 2% ketoconazole demonstrated comparable improvements in moderate-to-severe SD symptoms, with all subjects presenting only mild SD at that time. With a reduction of 71% for SeS_2_ and 69% for ketoconazole, the improvement was proven to be significant (*P* < 0.001)[6]. Studies have confirmed the usefulness of 2.5% SeS_2_ and 2% ketoconazole shampoos in soothing skin irritation, while 1% SeS_2_ may offer better cosmetic outcomes^[1,2]^.

Besides, the keratolytic activity of 1% SA found in SeS_2_-based shampoos promotes microbiome balance and reduces both adherent and nonadherent dandruff flakes^[2,5].^ Relapse rates in SD can be considerable, with about 49.5–50.5% of patients developing a relapse within 1 month of discontinuing treatment. Given this increased risk of recurrence, effective long-term management is crucial. Both SeS_2_ and zinc pyrithione are effective options, showing no difference in preventing recurrence or sustaining remission[7]. Existing research has demonstrated that SeS_2_ markedly decreases relapse rates compared with the vehicle, a trend that is further reflected in long-term maintenance outcomes. One of the findings showed that at week 6 – 4 weeks after stopping treatment with TCS/SA – the relapse rate in the SeS_2_-based shampoo group was 8.3%, which was five times lower than the 41.7% observed in the vehicle group, even with a once-weekly regimen. The relapse rate in the SeS_2_-based shampoo group decreased to 4.2% after 8 weeks of maintenance (*P* = 0.008), which is still three times lower than the vehicle group’s 12.5% rate. The SeS_2_ group’s long-term efficacy in maintaining remission was further supported by the fact that, overall, their maintenance relapse rate was almost four times lower (16.7%) than that of the vehicle group (54.2%)[2]. Research indicates that shampoos containing SeS_2_ are well tolerated at different application rates.

For the treatment of SD, a routine of two to three applications per week has been proven to be both well-tolerated and effective[3]. In another study, patients used SeS_2_ shampoo three times per week, leaving it on the scalp for 3–5 min before rinsing[7]. An intensive treatment schedule consisting of once-daily application for 3 consecutive days, followed by the same regimen 1 week later and maintenance therapy every 3 months, has also been reported as an effective approach[4]. In an RCT conducted post-ketoconazole treatment, patients applied SeS_2_ shampoo three times weekly, leading to improvements in dandruff and mild-to-moderate SD while positively influencing the scalp microbiome. Additionally, in the maintenance phase of the same study, patients initially treated with TCS/SA were assigned to either a once-weekly SeS_2_-based shampoo with a vehicle used twice weekly or a vehicle-only shampoo applied three times per week, both for 8 weeks[2]. The regimens above balance potency with patient adherence, showing that a less frequent treatment guideline may be helpful to prevent remission in many patients, improving treatment compliance and long-term management. A more recent study[5] has shown potential negative effects, such as scalp itching, irritation, and malodor[2]. Patient satisfaction is a key factor in following prescribed treatment, which may be influenced by dermatological acceptability and overall tolerability. A trial found that 92% of patients in the SeS_2_ group were satisfied with the product’s properties, and all found the shampoo easy to use, compared to 63% in the vehicle group[2].

Based on observations made earlier, future research should focus on optimizing SeS_2_-based formulations for sustained SD management and evaluating their efficacy in combination with other agents, particularly for severe or recurrent cases. The precise mechanism by which SeS_2_ impacts pruritus could be investigated. Research is required to evaluate how application frequency affects compliance. Understanding patient preferences and challenges can help refine treatment guidelines. SD manifestations vary by age, yet most SeS_2_-based research focuses on adults, leaving its efficacy, safety, and dosing in pediatric and geriatric populations rarely studied. Age-specific studies are needed for tailored treatments. Short-term studies may fail to capture critical outcomes, especially in mild cases, whereas long-term research is essential to evaluate its therapeutic potential, including sustained symptom alleviation comprehensively. Lastly, affordability and accessibility remain major factors in treatment success and quality of life. SeS_2_-based shampoos are widely available, making them a good choice, especially in resource-limited settings.

TITAN Guidelines: This manuscript complies with TITAN Guidelines, 2025, declaring no use of artificial intelligence^[8]^.

## Data Availability

Not applicable.

## Peer and provenance statement

Not commissioned, externally peer-reviewed.
